# Myeloperoxidase PET Imaging Tracks Intracellular and Extracellular Treatment Changes in Experimental Myocardial Infarction

**DOI:** 10.3390/ijms24065704

**Published:** 2023-03-16

**Authors:** Matthias W. G. Zeller, Cuihua Wang, Edmund J. Keliher, Gregory R. Wojtkiewicz, Aaron Aguirre, Kevin Maresca, Chunyan Su, Leonard Buckbinder, Jing Wang, Matthias Nahrendorf, John W. Chen

**Affiliations:** 1 Institute for Innovation in Imaging, Department of Radiology, Massachusetts General Hospital, Boston, MA 02129, USA; 2 Center for Systems Biology, Massachusetts General Hospital and Harvard Medical School, Boston, MA 02114, USA; 3 Pfizer World Wide Research and Development, Cambridge, MA 02139, USA; 4 Cardiology Division, Massachusetts General Hospital and Harvard Medical School, Boston, MA 02114, USA

**Keywords:** myeloperoxidase, PET imaging, ^18^F-MAPP, damaging inflammation, myocardial infarction, intracellular and extracellular MPO, MPO inhibitor

## Abstract

Myeloperoxidase (MPO) is a highly oxidative, pro-inflammatory enzyme involved in post-myocardial infarction (MI) injury and is a potential therapeutic target. While multiple MPO inhibitors have been developed, the lack of an imaging reporter to select appropriate patients and assess therapeutic efficacy has hampered clinical development. Thus, a translational imaging method to detect MPO activity non-invasively would help to better understand the role MPO plays in MI and facilitate novel therapy development and clinical validation. Interestingly, many MPO inhibitors affect both intracellular and extracellular MPO, but previous MPO imaging methods can only report extracellular MPO activity. In this study, we found that an MPO-specific PET imaging agent (^18^F-MAPP) can cross cell membranes to report intracellular MPO activity. We showed that ^18^F-MAPP can track the treatment effect of an MPO inhibitor (PF-2999) at different doses in experimental MI. The imaging results were corroborated by ex vivo autoradiography and gamma counting data. Furthermore, extracellular and intracellular MPO activity assays revealed that ^18^F-MAPP imaging can report the changes induced by PF-2999 on both intracellular and extracellular MPO activities. These findings support ^18^F-MAPP as a translational candidate to noninvasively report MPO activity and accelerate drug development against MPO and other related inflammatory targets.

## 1. Introduction

Myocardial infarction (MI) resulting from acute coronary events is one of the leading causes of morbidity and mortality worldwide. Percutaneous coronary interventions including angioplasty and stenting, thrombolysis, and coronary artery bypass grafting have significantly improved survival of patients [1], though secondary complications such as reperfusion injury, reinfarction, or heart failure occur frequently (up to 24% in recent studies [2,3]) after acute MI and further contribute to morbidity. Inflammation plays a crucial role in the pathophysiology of atherosclerotic plaque formation and rupture, and in the post-MI period [4]. With the onset of ischemia, inflammatory leukocytes, mainly neutrophils followed by monocytes, are recruited and infiltrated into the infarcted tissue [5]. These cells release proinflammatory cytokines, enzymes, and proteases that contribute to infarct expansion, ventricular dilatation, fibrosis, and eventually heart failure [4].

Among these, myeloperoxidase (MPO), a heme-containing and highly oxidative enzyme released by neutrophils and inflammatory monocytes, macrophages, and microglia, is a key marker of inflammation [6] and is involved in atherosclerosis and post-MI pathophysiology. MPO and its oxidative products contribute to the formation of foam cells, the hallmark of early-stage lesion formation in atherosclerosis, by oxidizing low-density lipoproteins and promoting endothelial dysfunction, apoptosis, plaque instability, and subsequent plaque rupture [7]. MPO mediates arrhythmogenic ventricular remodeling by inducing transdifferentiation of fibroblasts to myofibroblasts and promoting fibrosis post-MI [8]. MPO-generated oxidative products have a profound adverse effect on left ventricular remodeling and function [9]. Pharmacological inhibition or congenital absence of MPO decreased post-ischemic fibrosis, reduced left ventricular dilation, and improved left ventricular function in animal models of myocardial infarction [8,9,10,11]. Given the significant role it plays in MI and other inflammatory diseases, MPO has attracted substantial interest as a potential therapeutic target for anti-inflammatory treatment. Several MPO inhibitors have been developed, including PF-2999 for cardiovascular diseases [12,13]; AZD3241 for Parkinson’s disease [14]; PF-1355 for vasculitis, glomerulonephritis, and MI [10,15]; AZD4831 for heart failure (NCT03136991) [16]; and verdiperstat for multiple system atrophy (MSA) (NCT05086094) and amyotrophic lateral sclerosis (ALS) (NCT04436510), among others.

Molecular imaging approaches can provide a noninvasive tool to assess the progression of disease, monitor responses to novel treatments, and stratify patients to facilitate new treatment development. We have reported a myeloperoxidase-activatable PET probe (^18^F-MAPP) that not only detects MPO activity with high sensitivity and specificity but is also capable of monitoring damaging inflammation in mouse models of paw inflammation and myocardial infarction [17]. In an inflammatory environment, MPO uses hydrogen peroxide to oxidize the 5-hydroxy-indole units of the probe to form radicals that can reversibly bind to nearby tyrosine-containing proteins to be transiently retained at the site of activation and inflammation [17,18]. Interestingly, because MPO inhibitors are often small molecules, some of them (e.g., PF-1355 and PF-2999) can enter cells and act on intracellular MPO in addition to extracellular MPO [10]. However, none of the previous MPO-sensing imaging agents are known to enter cells to assess the effect of these MPO inhibitors on both intracellular and extracellular MPO activities. In this study, we aimed to demonstrate that ^18^F-MAPP can enter cells to detect not only extracellular MPO activity but also intracellular MPO activity to noninvasively track the therapeutic response of the specific MPO inhibitor PF-2999 in living animals induced with myocardial infarction.

## 2. Results

### 2.1. Radiochemistry

**Figure 1:**
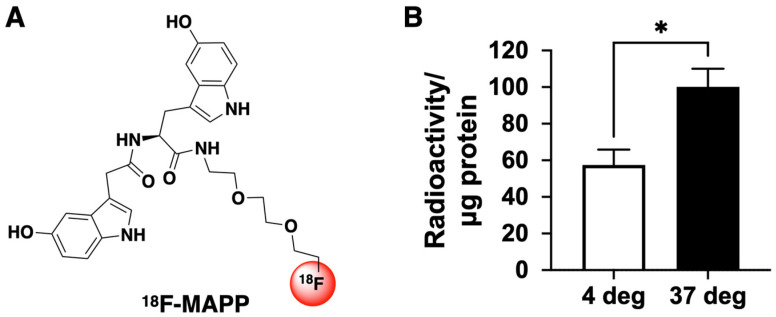
(**A**) Structure of ^18^F-MAPP. (**B**) ^18^F-MAPP can cross the cell membrane. After neutrophils were incubated with ^18^F-MAPP for 1 h and then washed to remove ^18^F-MAPP in the medium, radioactivity was still found in the neutrophils. Interestingly, the uptake was significantly higher at 37 °C than that at 4 °C, suggesting intracellular uptake may have both passive diffusion and energy-dependent components (* *p* < 0.05, *n* = 4, two-tailed *t*-test. Radioactivity was normalized to protein amounts obtained from BCA protein assay after gamma counting).

The automated synthesis and formulation of ^18^F-MAPP (Figure 1A) were performed as previously described [17].

### 2.2. ^18^F-MAPP Can Cross the Cell Membrane

We have previously demonstrated that ^18^F-MAPP can cross the blood–brain barrier (BBB) in vivo [17]. However, it was unknown whether ^18^F-MAPP can penetrate the cell membrane to detect intracellular MPO activity. To determine whether ^18^F-MAPP can penetrate the cell membrane, we incubated neutrophils from mouse bone marrow with ^18^F-MAPP for 1 h at 37 °C and 4 °C followed by washing to remove ^18^F-MAPP that had not entered into the cells. We found radioactivity in the cellular fractions at both 4 °C and 37 °C, revealing that the ^18^F-MAPP was able to enter into the cells at both temperatures (Figure 1B). Interestingly, ^18^F-MAPP cellular uptake at 37 °C was significantly higher than that at 4 °C, suggesting that ^18^F-MAPP cellular entry may have an energy-dependent mechanism in addition to passive diffusion.

### 2.3. ^18^F-MAPP Can Monitor the Treatment Effect of MPO Inhibitor PF-2999

**Figure 2:**
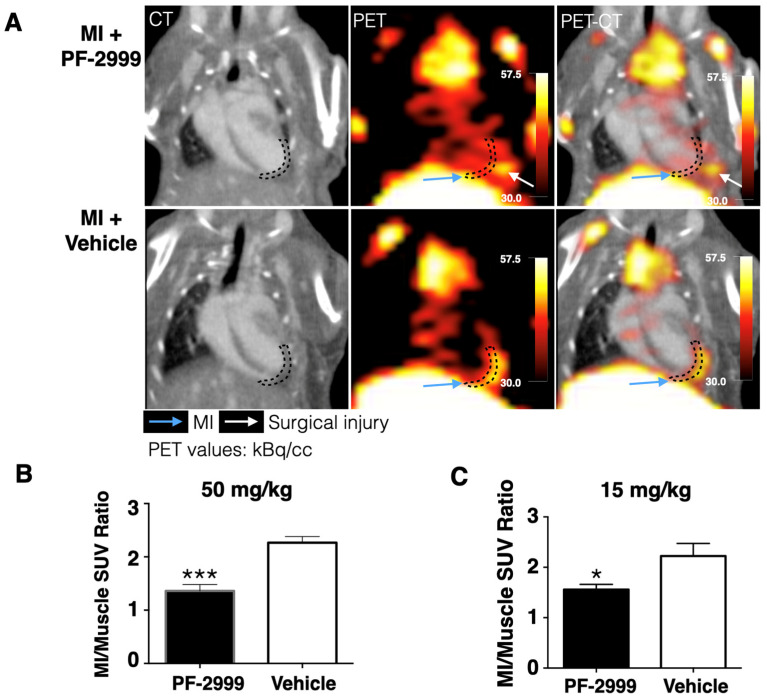
^18^F-MAPP imaging in a mouse model of myocardial infarction treated with PF-2999. (**A**) Representative CT, PET, and fused PET-CT images of MI mice treated with PF-2999 or vehicle (infarcted area outlined with dashed lines; blue arrow points to the infarct, and white arrow points to surgical injury). (**B**) ^18^F-MAPP uptake in mice treated with 50 mg/kg of PF-2999 showed a 40% reduction compared to that in the control group (*** *p* < 0.001, one-tailed *t*-test, *n* = 5 for the treatment group and 6 for control group). (**C**) ^18^F-MAPP uptake in mice treated with 15 mg/kg of PF-2999 showed a 30% of reduction versus that in the vehicle-treated group (* *p* < 0.05, one-tailed *t*-test, *n* = 4 for each group).

Inhibition of MPO activity can be a potential therapeutic strategy, and treatment with an MPO inhibitor has been found to improve ventricular function and remodeling in experimental MI [10]. We have shown that ^18^F-MAPP uptake was markedly higher in the infarcted myocardium compared to normal myocardium, verifying in vivo that MPO activity is elevated in the infarct [17]. We now use ^18^F-MAPP imaging to assess the efficacy of MPO inhibition in MI using an MPO inhibitor, PF-2999. PF-2999 is a selective and irreversible MPO inhibitor that covalently binds to and deactivates MPO, and its concentration curve has been previously published [12]. The IC_50_ of PF-2999 in inhibition of LPS-stimulated MPO activity in human whole blood is 1.9 µM [12] and treatment with a dose of 15 mg/kg led to a 73% reduction in MPO activity (normalized to MPO levels) in an atherosclerotic mouse model [13]. Another study using a similar inhibitor, PF-1355, in experimental myocardial infarction showed that a dose of 50 mg/kg was effective at improving outcomes [10]. Therefore, we chose a dose of 15 mg/kg and a higher dose of 50 mg/kg in this study. Mice with MI were treated with either 15 mg/kg or 50 mg/kg of PF-2999 or vehicle via oral gavage twice daily for 48 h after MI. The last dose was received 2 h before 700–800 µCi of ^18^F-MAPP was intravenously administered. PET-CT scanning was carried out 3 h after agent administration (Figure 2A). We observed a 40% reduction of ^18^F-MAPP uptake (presented as SUVR: standard uptake value ratio of infarcted area to skeletal muscles) in mice treated with 50 mg/kg of PF-2999 compared to that in control mice treated with vehicle (Figure 2B, *p* < 0.001, one-tailed *t*-test, *n* = 5 or 6 per group) and 30% uptake reduction in mice treated with 15 mg/kg of PF-2999 (Figure 2C, *p* = 0.025, *n* = 4 for each group, one-tailed *t*-test). These results confirmed that ^18^F-MAPP PET imaging can track treatment changes and monitor dose response (50 vs. 15 mg/kg) in MI when treated with an MPO inhibitor.

### 2.4. ^18^F-MAPP Imaging Correlates with Both Intracellular and Extracellular MPO Activities in MI

**Figure 3:**
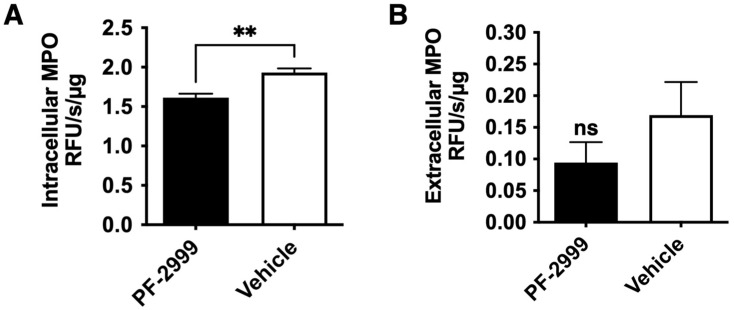
MPO activity assay with mice treated with 15 mg/kg of PF-2999. (**A**) Intracellular MPO activity in mice treated with PF-2999 was significantly lower compared to that in the control group (** *p* < 0.01, *n* = 4 for each group, one-tailed *t*-test). (**B**) Extracellular MPO activity in mice treated with PF-2999 showed 38% reduction compared to that in the control group (ns = not significant, *p* = 0.17, *n* = 4 for each group, one-tailed *t*-test).

In the previous study, we have shown that the ^18^F-MAPP signal was directly and linearly proportional to the amount of MPO activity embedded in Matrigel implants in a mouse thigh implantation experiment [17]. However, given that ^18^F-MAPP can enter cells (Figure 1B), we next examined for both extracellular and intracellular MPO activities in the infarcted myocardium when the animals were treated with either vehicle or 15 mg/kg of PF-2999 post MI. We found that there was a significant decrease in intracellular MPO activity in the infarcted myocardium treated with PF-2999 (16% reduction) compared to that treated with vehicle (Figure 3A, *p* = 0.002, one-tailed *t*-test, *n* = 4), and extracellular MPO activity was reduced by 38% in mice treated with PF-2999 compared to the control group (Figure 3B, *p* = 0.17, one-tailed *t*-test, *n* = 4). Note that the technique used to extract extracellular MPO [19] resulted in an unknown dilution factor, and thus we cannot directly compare the absolute amount of MPO activity between intracellular and extracellular experiments. Nonetheless, assuming equal contribution to the total MPO activity from both intracellular and extracellular MPO, we found an overall 27% reduction ([intracellular 16% + extracellular 38%]/2) in MPO activity with 15 mg/kg of PF-2999 treatment in the infarcted myocardium, which correlated well with the imaging result (30% reduction, Figure 2B).

### 2.5. ^18^F-MAPP Imaging Correlates with Ex Vivo Validation in MI

**Figure 4:**
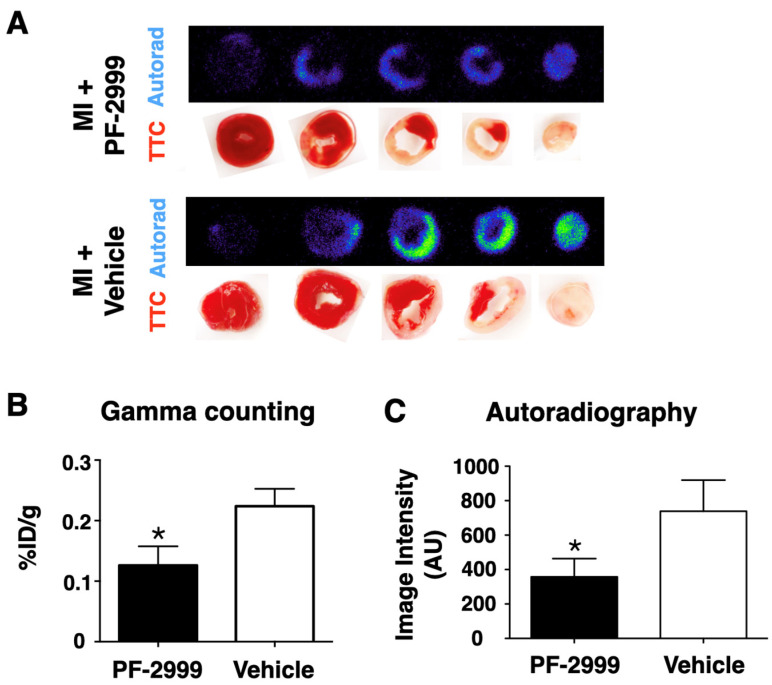
Ex vivo validation of ^18^F-MAPP uptake in hearts. (**A**) Autoradiography of the infarcted heart slices corresponded to the tetrazolium chloride (TTC) staining for both treated (with PF-2999, 50 mg/kg, top) and vehicle (bottom) groups, demonstrating that the probe is retained in the infarcted tissue after activation by MPO. Despite a comparable extent of the infarcted tissue in both groups as visualized by TTC staining, radioactivity was significantly lower in the heart slices from PF-2999-treated animals compared to those treated with vehicle, demonstrating the ability of ^18^F-MAPP to report pharmacological MPO inhibition in vivo. (**B**) Quantitative analysis by gamma counting (44% reduction with PF-2999, * *p* < 0.05, one-tailed *t*-test, *n* = 5 mice treated with PF-2999 and 6 with vehicle) and (**C**) quantitative analysis from autoradiography images (51% reduction with PF-2999, * *p* < 0.05, one-tailed *t*-test, *n* = 6 for each group). The ex vivo data from (**B**,**C**) corroborate the quantification of MPO activity and treatment-induced changes visualized in vivo on PET imaging in Figure 2.

To corroborate the imaging results, hearts from mice receiving 50 mg/kg of PF-2999 and vehicle were harvested after imaging. The heart tissue slices (1 mm) were subjected to gamma counting and autoradiography (Figure 4). Since PF-2999 treatment was administered after MI when irreversible ischemic tissue damage had already occurred, the triphenyltetrazolium chloride (TTC) staining of the heart slices demonstrated similar sizes of the infarcted tissue between PF-2999-treated mice and control mice (Figure 4A). However, the radioactivity was significantly lower in the heart slices from PF-2999-treated mice compared to those treated with vehicle. The total radioactivity of the heart slices in mice treated with PF-2999 demonstrated a 44% reduction compared to that in control mice (Figure 4B, *p* = 0.024, one-tailed *t*-test). This was matched by a similar reduction on autoradiography that showed a 51% reduction in PF-2999-treated MI mice compared to those treated with vehicle (Figure 4C, *p* < 0.05, one-tailed *t*-test). These ex vivo measurements correlated well to the imaging data (40% reduction), confirming the ability of ^18^F-MAPP imaging to report the pharmacological changes of MPO inhibition in vivo.

## 3. Discussion

In this study, we reported the application of ^18^F-MAPP in post-MI management and validated that ^18^F-MAPP imaging accurately reflected treatment changes with different dosages when treated with the MPO inhibitor PF-2999 (Figure 2B,C), corroborated by ex vivo autoradiography and gamma counting of the heart slices (Figure 4B,C).

Inflammation plays evolving roles after myocardial ischemia that influence ventricular remodeling, recovery, and function, and it has been recognized as a potential therapeutic target. The recruitment of leukocytes and consequent inflammation is indispensable to clearing debris and promoting healing and recovery [20,21]. However, an elevated and prolonged proinflammatory response from neutrophils and macrophages can impede the healing process [22]. An imaging method specific to beneficial or damaging inflammation would be highly useful in providing information on disease progression and monitoring the treatment response. The most commonly used PET imaging for inflammation is ^18^F-FDG due to elevated glucose metabolism of leukocytes during inflammation [23]. However, for myocardial infarction, ^18^F-FDG imaging is limited by the avidity of the cardiomyocytes for glucose [24]. In addition, ^18^F-FDG uptake reflects an elevated metabolic state, which is not specific to pro-inflammatory cells. Thus, imaging biomarkers that can specifically detect damaging inflammation and do not exhibit increased uptake in the normal myocardium would be highly desirable.

MPO is such a potential biomarker for damaging inflammation that has been found in neutrophils and Ly6C^high^ proinflammatory monocytes/macrophages but not in Ly6C^low^ reparative monocytes/macrophages [25]. As such, MPO has long been recognized as a marker in cardiovascular diseases. In addition to its contribution to atherogenesis [26,27], MPO is abundant in unstable atheroma and promotes plaque erosion and rupture in mouse models of atherosclerosis [13,28]. Elevated MPO levels in plasma are associated with adverse cardiovascular events, including future risk of coronary artery disease, endothelial dysfunction, and MI in humans [29,30,31]. As such, drugs targeting MPO have been developed. For example, MPO inhibition with the irreversible inhibitor PF-1355 decreased both extracellular and intracellular MPO activities, reduced left ventricular dilation, and improved cardiac function and scar remodeling in a mouse model of MI [10]. Another MPO inhibitor, PF-2999 [12] (an analog of PF-1355), was shown to be able to reduce subcutaneous inflammation [17]. In a mouse model of atherosclerotic plaque, MPO inhibition with PF-2999 was found to promote lesion stabilization and prevent plaque rupture [13]. Verdiperstat, another MPO inhibitor, is being developed for the treatment of neurodegenerative diseases such as multiple system atrophy (MSA) and amyotrophic lateral sclerosis (ALS). However, because damaging inflammation and oxidative stress generated by MPO are transient, without an in vivo reporter to select appropriate patients experiencing active damaging inflammation and to monitor therapeutic efficacy and target engagement, clinical studies testing these MPO inhibitors and other pro-inflammatory factors could be challenging. Imaging agents targeting MPO and its oxidative products covering multiple imaging modalities including MRI, PET, and fluorescence imaging have been investigated in the past two decades, and a comprehensive review on the progress of the MPO imaging agent development has been published recently [32]. These agents could be used to guide MPO inhibitor and anti-inflammatory therapy development in preclinical and clinical settings. However, until now, none of the existing MPO imaging agents have been capable of reporting intracellular MPO activity. We have found that ^18^F-MAPP can cross the intact BBB in a previous study [17], and in this study, we demonstrated that ^18^F-MAPP could also penetrate the cell membrane and contribute to the PET imaging signal (Figure 1B). While we only tested the cell membrane permeability of ^18^F-MAPP using inactivated neutrophils, considering that the major factors affecting cell membrane permeability are temperature and the properties of the permeant for non-charged small molecules in passive diffusion and transporter-mediated active transport [33], the slightly acidic surroundings of inflammation after neutrophil activation is not expected to significantly limit the permeability of ^18^F-MAPP, especially given that neutrophil activation would secrete factors that increase membrane permeability [34].

Although secreted extracellular MPO is considered a major contributor to damage and inflammation, intracellular MPO is only partially endogenously inhibited and could contribute to pathology, both by its role as a reservoir for secreted extracellular MPO or by its effects on the cells [35,36,37]. For example, MPO and its oxidative products can cause mitochondrial dysfunction and necrotic cell death to release proinflammatory signaling molecules to increase and propagate inflammation [38,39]. In addition, many drugs can also enter into cells. Indeed, we have previously found that both extracellular and intracellular MPO activities were significantly reduced when treated with 50 mg/kg of PF-1355, an analog of PF-2999 [10]. Similarly, our current study showed that intracellular and extracellular MPO activities in mice treated with 15 mg/kg of PF-2999 were reduced compared to those in control mice (Figure 3A,B), suggesting that both extracellular and intracellular MPO responded to these MPO inhibitors. Since ^18^F-MAPP can also enter cells, this agent allows for a more complete depiction of the overall MPO activity. Thus, both the intracellular and extracellular MPO activities contribute to the ^18^F-MAPP imaging signal, and our results confirmed that ^18^F-MAPP imaging is capable of highly accurate tracking of the treatment response in experimental MI by these drugs.

There are limitations in this study. Because we had previously reported a longitudinal study on the structural and functional outcomes from MPO inhibition in experimental MI [10], treatment and imaging of the myocardium were only performed during the acute stage (48 h) in this study. Future longitudinal research to study the evolution and treatment-related changes to intracellular and extracellular MPO activities and related cellular changes over the acute, healing, and chronic phases of MI combined with cardiac function monitoring could provide further insights into the progression of the disease and the involvement of MPO.

## 4. Materials and Methods

### 4.1. Chemicals and Radiochemistry

All chemicals were obtained from Sigma Chemical Co. unless otherwise stated. MPO inhibitor PF-2999 was kindly provided by Pfizer Inc, Cambridge, MA, USA.

^18^F-MAPP was prepared according to an established two-step synthesis as described in [17] using an automated synthesizer (Synthra RNplus, Synthra, GmbH, Hamburg, Germany).

### 4.2. Cell Penetration

Neutrophils were extracted from mouse bone marrow and separated with magnetic negative isolation as follows. Femur and tibia bone shafts were flushed with a 25 G needle and a 10 mL syringe containing MACS buffer (phosphate-buffered saline (PBS) + 1% fetal bovine serum (FBS) + 0.2% EDTA). The bone marrow was homogenized, filtered through a 40 µm cell strainer (BD Biosciences, San Jose, CA, USA), and centrifuged at 400 G for 7 min. The pellet was resuspended in red blood cell (RBC) lysis buffer (Biolegend, San Diego, CA, USA) and incubated for 3 min. Cells were diluted, centrifuged, and resuspended in 500 µL of MACS buffer, stained with a cocktail of PE-antibody of CD220/CD19/CD90/CD4/CD8/c-kit/Ter119 (3 μL for each) in the dark for 20 min. Then the cells were diluted, centrifuged, resuspended in 500 µL of MACS buffer, and incubated with 60 µL of anti-PE beads for 15 min at 4 °C. Cells were washed, resuspended, and separated with magnetic LS columns (Miltenyi Biotec, Bergisch Gladbach, Germany) to collect the magnetic negative cells. Then 300–600 μCi of ^18^F-MAPP in saline was added to the neutrophils (~1.4 x10^6^) in a 96-well plate and incubated for 1 h at 4 °C or 37 °C (*n* = 4). Then the cells were washed 3 times with PBS, and the radioactivity was assayed with a gamma counter (1480 Wizard 3, PerkinElmer, Waltham, MA, USA). The BCA protein assay (Thermo Scientific Pierce^TM^ BCA assay kit, Waltham, MA, USA) was performed the following day to determine protein concentration.

### 4.3. Myocardial Infarction (MI) and Treatment with PF-2999

Six to ten weeks old female C57BL/6J mice (Jackson Laboratories, Bar Harbor, ME, USA) were used for the animal experiments. Myocardial infarction was induced by permanent occlusion of the left anterior descending artery. Briefly, after being anesthetized by isoflurane, shaved and disinfected with 70% isopropanol followed by betadine, the mouse was intubated using a laryngoscope and a 22 G angiocatheter sheath and ventilated with a small animal ventilator (Model 683, Harvard Apparatus, Holliston, MA, USA). A thoracotomy was performed between the 3rd and 4th intercostal space. After removing connective tissue from the heart, an 8-0 monofilament suture was put through the upper aspect of the left ventricle to ligate the left coronary artery. Two separate 5-0 monofilament sutures were made to close the rib cage, a 20 G angiocath chest tube was left between the sutures, and the overlying muscle layer was sutured in an interrupted fashion with 6.0 silk suture. Air was removed from the thoracic cavity by gentle suction on the chest tube, and the tube was subsequently removed. The overlying skin layer was then sutured closed with a running continuous suture of 6.0 silk. The mice were subsequently randomized to receive either 50 mg/kg or 15 mg/kg of the MPO-inhibitor PF-2999 or vehicle twice daily via oral gavage. Then, 48 h after myocardial infarction and treatment, animals were subjected to the following imaging, followed by ex vivo autoradiography and MPO activity assay.

### 4.4. PET-CT Imaging

Six to ten weeks old female C57BL/6J mice (Jackson Laboratories, Bar Harbor, ME, USA) were used for all animal experiments. PET imaging was performed on a Siemens Inveon PET-CT scanner. Then 700–800 μCi of ^18^F-MAPP was intravenously injected while the mice were under anesthesia (~2% isoflurane in oxygen). After a washout period of 3 h, Isovue 370 iodine contrast was pumped into the mice via the tail veins at 40 μL per minute for the duration of the 5 min whole-body CT scan, followed by a 30 min static PET scan under respiratory monitored isoflurane anesthesia. The CT X-ray source used an 80 kVp and 500 μA tube with an exposure time of 425 ms over 360 projections and reconstructed by a modified Feldkamp conebeam reconstruction algorithm (COBRA, Exxim Inc., New York, NY, USA) into isotropic 110 μm voxels. The PET data were reconstructed with 3D ordered subset expectation maximization with maximum a posteriori with a spatial resolution of approximately 1.5 mm. Volumes of interest (VOIs) were manually drawn for standard uptake value (SUV) quantification of the images in Inveon Research Workplace. Image visualization was performed using Horos (horosproject.org, accessed on 20 February 2021).

### 4.5. Autoradiography

After PET-CT imaging, mice were sacrificed by trans-cardiac perfusion with 20 mL of ice-cold PBS under deep anesthesia, and hearts were harvested for gamma well counting and autoradiography. Mice hearts were sliced into 1 mm thick slices followed by triphenyltetrazolium chloride (TTC) staining and then placed on a storage phosphorimager plate for approximately 14 h. Then the slices were imaged on a Typhoon 9410 scanner (GE Healthcare).

### 4.6. MPO Activity Assay

Both extracellular and intracellular MPO activity assays were performed according to an established protocol [19]. Briefly, after imaging, the infarcted myocardium was isolated from the hearts and washed with PBS. The infarcted myocardium was incubated with extraction buffer (0.32 M sucrose, 1 mM CaCl_2_, 10 U/mL heparin in HBSS) of 4 times the organ weight for 2 h on ice. The extraction buffer containing extracellular proteins was centrifuged for 5 min at 500 G at 4 °C to remove loose tissue debris and cells and went through a 50 kD MWCO filter (Amicon^®^, Burlington, MA, USA) to obtain concentrated extracellular proteins for the MPO activity assay. The infarcted myocardium (stored on ice) from the previous step was homogenized with 500 µL of CTAB buffer (50 mM potassium phosphate at pH 6.0 with 50 mM CTAB) using a mechanical tissue homogenizer for 30 s, then sonicated in a water-bath sonicator for 30 s; samples were freeze–thawed in liquid nitrogen. The samples were centrifuged at 15,000 G for 15 min at 4 °C and the supernatants containing intracellular proteins were obtained for MPO activity assays. BCA assays were performed to obtain protein concentrations per sample, and based on this, the samples were diluted at 1:2 to 1:20 with dilution buffer (Hycult). Then 100 μL of each diluted sample was added to the MPO activity assay plates (Hycult) coated with MPO–antibody on the well bottom and incubated for 1 h at room temperature. Wells with PBS only or isolated bone marrow-derived neutrophils were used as a negative or positive control, respectively. The wells were washed 4 times with 300 μL washing buffer (PBS with 0.05% Tween-20). Then 1 µL 0.03% H_2_O_2_ in 49 µL PBS was added to each well followed by adding 50 µL of ADHP working solution (200 mM ADHP in DMSO diluted in 1:1000 in PBS). Fluorescence in kinetic mode was immediately recorded using a microplate reader (Tecan Safire2, Tecan, Switzerland) for 5 to 10 min with excitation at 535 nm and emission at 590 nm. The MPO activity was calculated by using linear regression to obtain the slope value.

### 4.7. Statistical Analysis

Statistical analysis was performed using Prism 9.3 (GraphPad, La Jolla, CA, USA). Data were shown as mean ± SEM. Most of the data were compared using the one-tailed *t*-test. Linear regression was performed for the BCA protein assays. Normality of the data was tested using the Shapiro–Wilk test. The two-tailed *t*-test was used to compare the degree of ^18^F-MAPP cellular uptake at different temperatures, and *p* values < 0.05 were considered statistically significant.

## 5. Conclusions

In summary, we showed that ^18^F-MAPP imaging can accurately report the treatment changes with MPO inhibition, including contributions from both intracellular and extracellular compartments, and is sensitive to different drug dosages in experimental MI. The high sensitivity and specificity of ^18^F-MAPP to MPO activity make it a promising candidate for detecting damaging inflammation. Translation of ^18^F-MAPP imaging is underway.

## 6. Patents

J.W.C., C.W., and E.J.K are inventors of a patent based on the technology reported in this manuscript (US Patent 11,352,326).

## Data Availability

The data presented in this study are available within the article.
